# Combining printing and nanoparticle assembly: Methodology and application of nanoparticle patterning

**DOI:** 10.1016/j.xinn.2022.100253

**Published:** 2022-04-27

**Authors:** Weidong Zhao, Yanling Yan, Xiangyu Chen, Tie Wang

**Affiliations:** 1 National Engineering Research Center for Advanced Polymer Processing Technology, College of Materials Science and Engineering, Henan Province Industrial Technology Research Institute of Resources and Materials, Key Laboratory of Advanced Material Processing & Mold (Ministry of Education), Zhengzhou University, Zhengzhou 450002, China; 2 Beijing National Laboratory for Molecular Sciences, Key Laboratory of Analytical Chemistry for Living Biosystems, Institute of Chemistry, Chinese Academy of Sciences, Beijing 100190, China; 3 Life and Health Research Institute, School of Chemistry and Chemical Engineering, Tianjin University of Technology, Tianjin 300384, China

**Keywords:** nanoparticles, self-assembly, printing technology, patterned structure, functional devices

## Abstract

Functional nanoparticles (NPs) with unique photoelectric, mechanical, magnetic, and chemical properties have attracted considerable attention. Aggregated NPs rather than individual NPs are generally required for sensing, electronics, and catalysis. However, the transformation of functional NP aggregates into scalable, controllable, and affordable functional devices remains challenging. Printing is a promising additive manufacturing technology for fabricating devices from NP building blocks because of its capabilities for rapid prototyping and versatile multifunctional manufacturing. This paper reviews recent advances in NP patterning based on the combination of self-assembly and printing technologies (including two-, three-, and four-dimensional printing), introduces the basic characteristics of these methods, and discusses various fields of NP patterning applications.

## Public summary

• Nanoparticles (NPs) printing assembly is a good solution for patterned devices
• NPs assembly can be combined with 2D, 3D, and 4D printing technologies
• A variety of ink-dispersed NPs are available for printing assembly
• NPs printing assembly technology is applied for nanosensing, energy storage, photodetector

## Graphical abstract

**Figure:**
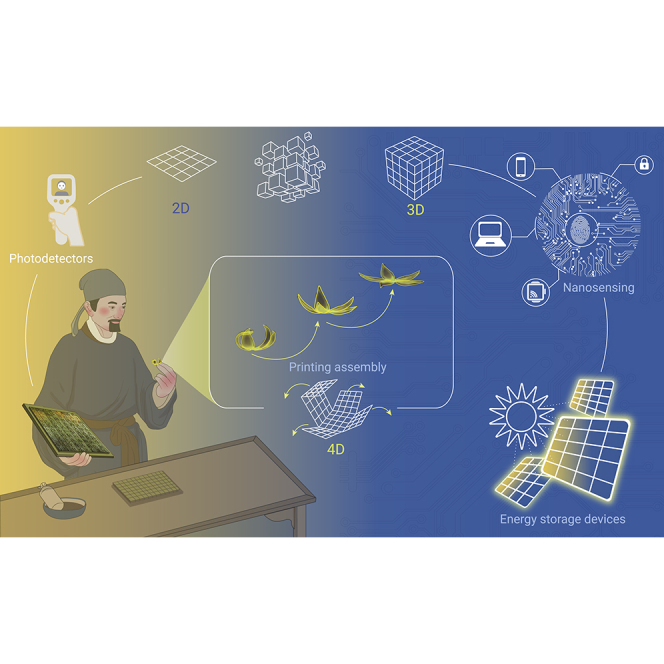

## Introduction

Functional nanoparticles (NPs) have attracted attention because of their unique physicochemical properties, such as the quantum confinement effects of quantum dots (QDs),^1^ superparamagnetism of magnetic NPs,^2^^,^^3^ and surface plasmon resonance (SPR) of metal NPs.^4^^,^^5^ The designed aggregation of NPs provides collective optical, electrical, and magnetic properties, which are different from those of discrete NPs.^6^, ^7^, ^8^ The assembled functional NPs are transformed into functional devices for specific applications, such as ultrasensitive sensing and integrated circuit design. These applications require tailoring the two-dimensional (2D) patterns, three-dimensional (3D) architectures, or four-dimensional (4D) dynamically transformed structures of custom-assembled NPs. Traditional thin-film manufacturing methods, such as casting, Langmuir-Blodgett, and doctor blading, only enable NPs be stacked in disorder.^9^, ^10^, ^11^ Printing technologies can perform elaborate patterning and well-defined positioning.^12^, ^13^, ^14^ Therefore, a hybrid strategy combining NP assembly and printing processes can adequately overcome this challenge. The advantage of this strategy is that it allows functional NPs with different controlled shapes, thicknesses, resolutions, and layouts to maximize their potential for specific applications. Thus, printing assembly is not only a tool for patterning but also a support tool for new applications and industrialization. NP patterning has been employed in nanosensing, energy-storage devices, and photodetectors. For example, plasmonic NPs are generally used as Raman, infrared, and fluorescence amplification materials to recognize biological proteins and cancer cells.^15^^,^^16^ Metal oxide NPs have been used as energy conversion materials to greatly improve the photoelectric conversion efficiency of solar cells.^17^

**Figure 1:**
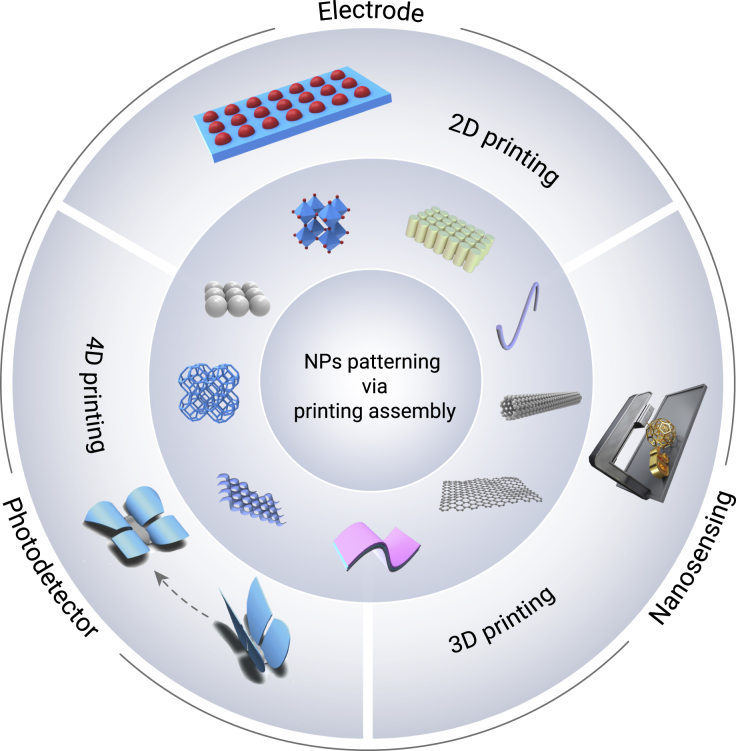
Schematic Illustrating the NP patterning via printing assembly: preparation method and its application

This review focuses on recent advances in various fabrication methods for forming large-area NP patterning based on 2D, 3D, and 4D printing assemblies (Figure 1). The advantages and disadvantages of parallel printing assembly techniques for NPs are evaluated and summarized. Additionally, nanoinks based on different nanomaterials have been used for printing assemblies. Finally, the applications of NP patterning produced using printing assembly technology are reviewed. This review aims to deepen the understanding of the printing assembly technologies of functional NPs and to provide new ideas for multidisciplinary research.

### NP assembly based on 2D printing

The assembly units of NP building blocks have enabled the implementation of on-demand high-resolution patterning by 2D printing assembly on a variety of substrates, such as 2D, 3D, rigid, and flexible substrates.^18^, ^19^, ^20^ The 2D printing methods are divided into template-based and nozzle-based printing. Nozzle-based printing assembly is based on inkjet printing, which is a non-contact, high-resolution, maskless NP patterning technology.^21^^,^^22^ Template-based printing assembly is mainly based on screen printing,^23^^,^^24^ nanoimprinting (NIL),^25^, ^26^, ^27^ microcontact printing (μCP),^28^^,^^29^ and evaporative lithography printing^30^^,^^31^ for low-cost and large-scale manufacturing. This section comprehensively discusses the principles and mechanisms of these printing assembly methods.

#### Inkjet printing assembly

**Figure 2:**
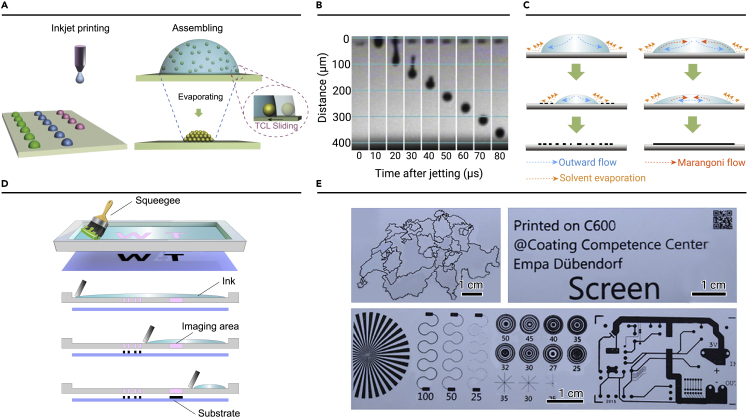
Inkjet printing assembly and screen-printing assembly method for NPs (A) Schematic diagram of the inkjet printing assembly process. (B) Time-sequence image of ink droplet ejection. (C) Schematic diagram of microdroplets drying with and without cyclic Marangoni flow induction. (D) Schematic diagram of the screen-printing assembly process. (E) Optical photographs of the constructed devices, including miniature supercapacitors, conductive rails, integrated circuits. Reprinted with permission from Kuang et al.^32^ (copyright 2014, John Wiley & Sons) (A), Hu et al.^38^ (copyright 2017, Nature Publishing Group) (B and C), and Abdolhosseinzadeh et al.^39^ (copyright 2020, John Wiley & Sons) (E).

Inkjet printing assembly is a simple, fast, and universal technology for the formation of microscale high-resolution patterns with a variety of nanomaterials.^32^ The NP ink is extruded into tiny nozzles by voltage-controlled pressure, and then naturally drops onto the substrate.^33^ Then, the ink droplets dry on the substrate and the NPs are assembled (Figure 2A). This technology enables a low-cost and efficient arrangement of droplets on the microscale, allowing precisely controlled deposition of small amounts of NPs. Moreover, owing to its advantages of arbitrary design and large-scale preparation, this technology has become a widely used method for manufacturing high-performance electrodes and multi-channel sensors.^34^^,^^35^ Many challenges still exist in controlling the quality of the printed pattern, including the viscosity and surface tension of the NP ink and the wettability of the printing substrate.^36^ The nozzle diameter is generally 10–30 μm. To avoid clogged nozzles, the diameter of the NPs should not be larger than that of the nozzle. In order to obtain an accurate NPs pattern, it is necessary to adjust the ink properties so that the ink drops drop vertically at the specified position (Figure 2B).^33^ The inverse Ohnesorge number, Z, is commonly used to characterize the fluid properties and ink printability, as expressed in Equation 1:^37^

(Equation 1) $$Z=\frac{\alpha }{\sqrt{\sigma \rho d}}$$

where *α* is the fluid viscosity, *d* is the nozzle diameter, *ρ* is the fluid density, and *σ* is the surface tension. Jang et al. suggested that the best range for Z in inkjet printing is 4–14. Another important factor is the wettability of the substrate, which significantly controls the spread of ink droplets on the substrate, thus regulating the assembly behavior of the NPs. Generally, hydrophilic substrates are conducive to the self-assembly of NPs into ordered structures.

The ink-drying process is critical for the uniform deposition of NPs after printing. When ink droplets containing suspended NPs dry on hydrophilic substrates, the suspended NPs gather and deposit along the edges of the original droplets as the water-based ink spreads over them, leading to the coffee-ring effect.^40^ This phenomenon is caused by the flow imbalance of the droplet system during drying. The higher surface-to-volume ratio at the droplet edge causes the solvent to evaporate faster than at the droplet center, resulting in an outward flow from the center to the edge, carrying the dispersed NPs to the fixed three-phase contact line (TCL).^41^ Previous studies described inks composed of binary solvent mixtures that produce different evaporation rates. Fast-evaporating solvents have the highest proportion in the center, while slow-evaporating solvents are highest in proportion at the edges, resulting in surface tension driven (Marangoni) recirculating flows.^38^, ^42^, ^43^ This flow can prevent the formation of coffee rings and ensure the uniform deposition of NPs, as shown in Figure 2C.^38^ Furthermore, the shape of the NPs, surface tension and viscosity of inks, and ambient temperature have an important influence on the formation of the coffee ring. Yunker et al. proposed that elliptic NPs can deform the interface and produce a strong capillary interaction between NPs.^44^ Song et al. further discovered that the free shrinkage of NPs is promoted on low-viscosity and superhydrophobic substrates.^45^

The advantages of inkjet printing include (1) that the deposition process does not use masks, which facilitates the printing of complex patterns; (2) various substrates, such as rigid silicon, flexible polymer, and even commercial paper substrates, can be used for printing; and (3) the high-resolution output shows potential for manufacturing miniature devices. The main disadvantage of inkjet printing is the difficulty of preparing printable inks with appropriate rheological properties.

#### Screen-printing assembly

Screen-printing assembly refers to the uniform spread of NP ink on the substrate to form an NP pattern using a screen as a template.^46^ This process usually involves three steps (Figure 2D); namely, the ink is evenly painted onto the substrate using a screen; the ink penetrates the screen window and laminates on the target substrate; and the screen template is removed after the ink dries. Plate and roll-to-roll printing are the two main screen-printing modes. Zhang et al. used screen-printing technology to print NPs ink containing multiple layers of MXene to prepare devices, including miniature supercapacitors, conductive rails, and integrated circuits, as shown in Figure 2E.^39^ Roll-to-roll printing presses ink onto a substrate through a polyester mesh cylinder and perforated metal. This technology can be used to produce numerous identically patterned arrays in one step. Ko et al. demonstrated a flexible all-solid supercapacitor with a flexible silver NP current collector.^47^ The ink should have a certain viscosity to prevent ink leakage on the design screen template.^48^ The thickness of the pattern (d; μm) produced by screen printing is mainly controlled by the window area of the screen (A; m^2^); deposition yield (K_d_); and the density (ρ; kg/m^3^), concentration (c; kg/m^3^), and volume (V; mL) of the ink, as expressed in Equation 2:

(Equation 2) $$d=k_{d}\left(\frac{V}{A}\right)\left(\frac{c}{\rho }\right)$$

where K_d_ is determined by the blade velocity, ink viscosity, and force on the screen.^37^ The consistency of the printing process should be maintained to repeatedly obtain a uniform film.

The advantages of screen-printing assembly are its low cost and high speed. The deposition rate is considerably higher than that of other printing technologies. Additionally, the preparation process is highly compatible with the working environment, which is not limited by the material, shape, or size of the NPs (polymer, metal, inorganic non-metallic, metal organic framework [MOF] NPs, and others) and can be printed on special substrates such as curved surfaces or spheres. However, the disadvantages of screen-printing assembly are its low resolution, high roughness, and the need for high-viscosity ink.

#### NIL assembly

**Figure 3:**
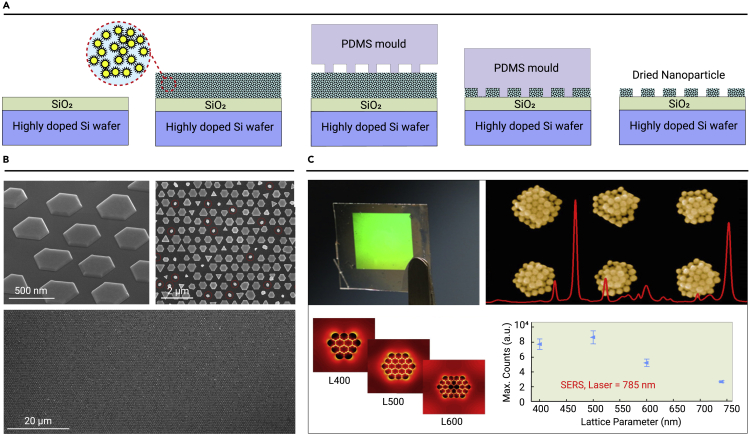
NIL assembly method for NPs (A) NIL preparation process for NP self-assembly. (B) Top-view, tilted-view, and low-magnification SEM images of the Au nanoplate array constructed by NIL. (C) photographs, SEM images, and SERS performance of AuNP arrays based on a soft PDMS mold. Reprinted with permission from Ko et al.^26^ (copyright 2007, American Chemical Society) (A), Golze et al.^50^ (copyright 2019, American Chemical Society) (B), and Matricardi et al.^52^ (copyright 2018, American Chemical Society) (C).

NIL is a novel micro/nanoprocessing technology. The resolution can reach the micrometer or submicrometer level through mechanical transfer. It is expected to replace traditional lithography technology and become an important means of processing microelectronics and materials. The NIL preparation process for the assembly of NPs is shown in Figure 3A. The NIL assembly typically drops NP ink onto the substrate, which is then pressed onto the ink using a rigid pattern template. As the solvent evaporates, the NPs are assembled to form a pattern.^26^^,^^49^ Neretina et al. reported a hybrid strategy based on NIL and gas-phase assembly to form periodic seed arrays, which were driven by liquid-phase plasma mediation and photostimulation to produce periodic arrays of hexagonal gold nanosheets (Figure 3B).^50^ Kraus et al. directly nanoimprinted metal nanowires as a conductive metal mesh to produce conductive materials with adjustable properties. The mechanical properties were significantly better than those of commercial indium tin oxide.^51^

NIL assembly is a powerful tool for achieving elaborate patterns. However, residual NPs can remain on the template after processing, resulting in pattern defects. A polydimethylsiloxane (PDMS)-based elastomer stamp template is used to remove structural defects. Liz-Marzan et al. reported that the PDMS template-assisted assembly of gold NPs (AuNPs) realized hexagonal stacked supercrystals with a period of 400 nm and an area of 0.5 cm^2^ (Figure 3C).^52^^,^^53^ These regular 2D supercrystals exhibited well-defined collective plasmon modes that could be tuned from visible to near-infrared (NIR) by simple changes in the lattice parameters.

NIL assembly has the following advantages: (1) the preparation process is simple and inexpensive; (2) ordered NP patterns can be generated over large areas or transferred to various substrates (planar, non-planar, flexible, or rigid); and (3) functional nanostructures can be customized by selecting NPs of different types, materials, and sizes. Murray et al. reported hybrid nanorods consisting of superparamagnetic Zn_0.2_Fe_2.8_O_4_ and AuNPs by NIL assembly, with both superparamagnetic and plasma characteristics. The combination of superparamagnetic and plasmon properties can switch the infrared transmission of the mixed nanorods suspension by the application of an external magnetic field.^54^ The main disadvantage of NIL assembly is the need for primitive mother templates with nanoscale dimensions and specific shapes, which generally require inefficient and expensive top-down lithography methods. To solve this problem, ordered nanostructures have been successfully used to fabricate NIL templates. Anodic alumina films with hexagonal nanopore arrays are an attractive alternative.^55^ Additionally, the mother template is usually used less than 50 times and has a short life.

#### μCP assembly

**Figure 4:**
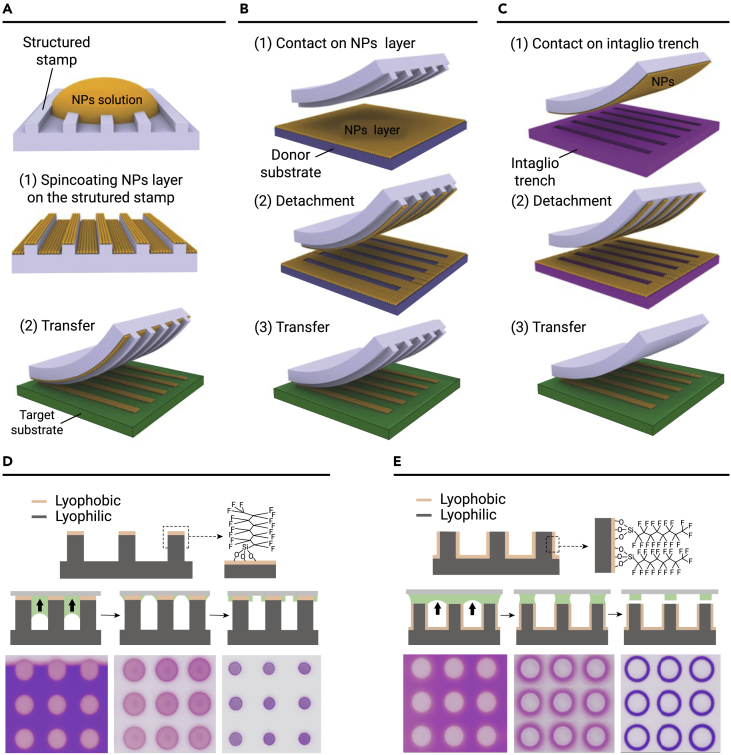
μCP assembly and evaporative lithography assembly for NPs (A–C) Schematic diagram of the three different types of μCP assembly of additive transfer printing, subtractive transfer printing, and intaglio transfer printing. (D and E) Two methods of evaporative lithography assembly of capillary-trailing manipulation and capillary-bridge manipulation. Reprinted with permission from Yang et al.^37^ (copyright 2015, John Wiley & Sons) (A–C), and Geng et al.^60^ (copyright 2020, Acta Polymerica Sinica) (D and E).

μCP is a micromachining technique for picking and placing NPs onto a target substrate to form a pattern using a soft elastic stamp.^56^ The combination of μCP and self-assembly technology can achieve a patterned NP array.^57^ The technology can be processed on different types of target substrates, making this a room-temperature, simple, and low-cost process. μCP assembly can be divided into three types according to the transfer mode, namely additive, subtractive, and intaglio transfer, as shown in Figures 4A–4C. To transfer the NP array to the target substrate, the adhesion work between the PDMS/NP interface (W_12_) and the NP/target substrate (W_23_) requires that W_12_ > W_23_. The adhesion work can be calculated using Equation 3:^35^

(Equation 3) $$W_{12}=4\left(\frac{\gamma_{1}^{d}\gamma_{2}^{d}}{\gamma_{1}^{d}+\gamma_{2}^{d}}\right)+\left(\frac{\gamma_{1}^{p}\gamma_{2}^{p}}{\gamma_{1}^{p}+\gamma_{2}^{p}}\right)$$

where *γ* is the surface free energy (kg m^−1^ s^−2^) and *d* and *p* represent the dispersion and polarity components of *γ*, respectively. The NP array can be easily transferred from the stamp to the target substrate when the PDMS stamp surface energy is significantly lower than that of the target substrate. It is worth noting that the quality of NP self-assembly determines the quality of the final pattern during the transfer process. Alexander et al. first assembled AuNPs on a soft PDMS template by electrostatic interaction.^58^ Then, poly(ethylene imine) surface-decorated wrinkled stamps were employed to contact the template to form gold nanolattices. Owing to the independent tunability of the orientation between the stamp and the target substrate, NP arrays with different geometric shapes could be directly formed. μCP assembly has been successfully demonstrated in several practical applications. Nanocube dimers were obtained using the μCP method. The surface-enhancement Raman spectroscopy (SERS) signal was significantly enhanced by this structure.^59^

The advantages of this technology, such as low cost, ease of operation, universality, and lower costs due to material savings, have stimulated interest in creating high-resolution patterns. However, most stamps consist of hydrophobic PDMS, which is easily swelled by polar solvents and cannot be wetted by water-soluble inks. Additionally, any distortion of the stamp affects the printed pattern and reduces reproducibility. These problems limit the application scope of μCP.

#### Dip-pen assembly

Dip-pen nanolithography (DPN) is a technology based on atomic force microscope (AFM) for direct writing with cantilevers.^61^ The technology has high resolution (nanometer level) and can be combined with NPs (such as metals, colloid, organic, semiconductor, and biological) to assemble and construct high-performance patterned arrays.^61^, ^62^, ^63^ The DPN preparation process for NP assembly is generally divided into four steps, namely dip coating, solvent evaporation, humidity adjustment, and assembly patterning.^60^ First, the NP ink is coated on the AFM tip and transmitted through the meniscus to the substrate. The contact interface between the tip and substrate forms a condensed water meniscus, which is regarded as the NP ink transfer channel. The NPs are then patterned by a computer, which controls the motion of the tip.^64^ The pattern quality for NP assembly is mainly controlled by the transport process, which is generally divided into three stages: (1) dissolution, in which the NP ink is dissolved at the tip and is transferred to the meniscus. The activation energy of the meniscus controls the dynamics of the NP transport process, and the ink coverage on the tip determines the transfer rate of the NP ink;^65^^,^^66^ (2) diffusion, in which the NP ink on the AFM tip diffuses toward the substrate through the bending liquid. Peterson et al. reported that the NP ink diffusion rate increases with an increasing ambient humidity and is negatively correlated with the lithographic area in DPN;^67^^,^^68^ and (3) assembly, in which NP inks exhibit capillary force, surface tension, Laplace pressure, and chemical reaction-driven assembly molding. For example, Biswas et al. assembled patterned CdSe/ZnS colloidal NPs with DPN.^69^ Phospholipids and cholesterol were added to the NP ink as the transfer medium. The NP ink was assembled under the interaction of Laplacian forces and surface tension to obtain a high-resolution (4 nm) NP composite pattern. Moreover, Saha et al. proposed a model capable of directly predicting feature sizes from parameters such as the meniscus, tip, and surface.^70^

DPN assembly technology has a high resolution, low cost, and a wide range of inks. Moreover, DPN assembly can be combined with polymer pen lithography and beam pen lithography cantilevered techniques to fabricate many types of NP structures owing to the multiplex and high-throughput preparation.^71^, ^72^, ^73^ Although DPN technology has made good progress, some challenges still remain. For example, the spatial resolution of DPN-assembled NP patterns is limited by the meniscus surface, curvature radius of the tip, and the nanoreactor. Moreover, the printing area is usually small.^62^

#### Evaporative lithography assembly

Evaporative lithography assembly is a method for fabricating well-defined nanostructures induced by capillary forces. Vakarelski et al. first reported a nanosphere array as a template to guide AuNPs to form a liquid-bridge network between the nanospheres and substrate.^74^ The AuNP patterned scale is controlled by surfactants. Furthermore, a well-controlled method for rectangular, honeycomb, and hexagonal topological structures was developed using a cylindrical top arch template.^75^ Song et al. investigated the effect of regulating the wettability of patterned substrates using evaporative lithography assembly. The droplets wet the entire microcolumn on the high-viscosity hydrophilic silicon substrate and exhibited a typical Wenzel state. After the liquid gradually decreased, NPs were deposited on the top and side of the column. The droplets displayed a non-wetting state on the superhydrophobic microcolumn, and the NP droplets only adhered to the top of the substrate, showing a Cassie state. Droplets tend to evaporate and crystallize at the top of the column on a highly viscous substrate; therefore, superhydrophobic substrates with high adhesion were used as candidate substrates for pattern preparation. To meet a wide range of applications, the obtained nanostructures are generally transferred to the desired substrate. Wu et al. developed a sandwich-shaped system that directly prints nanopatterns onto a desired substrate.^76^ The system consists of a microcolumn template, NP solution, and target substrate. When the top of the microcolumn is hydrophobically modified, the droplets gather at the side wall of the microcolumn to further spread out, finally forming a microstructure at the microcolumn edge (Figure 4D).^77^ When the side wall of the microcolumn is hydrophobic, the liquid film does not permeate between the microcolumns because the side wall is superhydrophobic, anchoring the liquid bridge at the top of the microcolumn, and finally forming a microstructure at the top of the microcolumn (Figure 4E). The liquid transport of droplets on traditional substrates occurs mainly through an isotropic random capillary flow to a TCL, resulting in the prepared structure having low crystallinity. The discrete capillary bridge during the printing process allows directional transport of the liquid to the TCL, thus obtaining high-quality crystal arrays.

Evaporative lithography assembly is similar to NIL and μCP assembly in that nanotemplates are used to assemble NPs into desired pattern arrays. Therefore, the advantages and disadvantages of this technique are consistent with those of these two methods.

2D printing assembly provides a simple, flexible, and cost-effective solution for the rapid manufacture of functional devices. However, printing large 3D devices using traditional 2D printing techniques remains a challenge.

### NP assembly based on 3D printing

**Figure 5:**
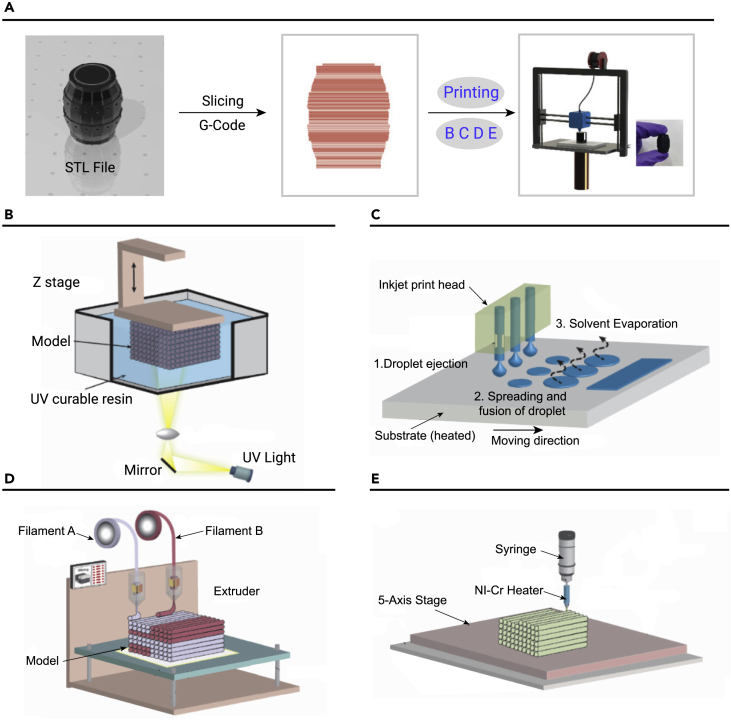
3D printing assembly for NPs (A) Schematic diagram of the 3D printing process, which generally involves modeling, slicing, printing. (B–E) The main methods to implement 3D printing assembly include (B) SLA, (C) DIW printing, (D) FDM printing, (E) EHD printing. Reprinted with permission from Xu et al.^81^ (copyright 2021, John Wiley & Sons) (A–E).

3D printing is a new additive printing technology.^78^, ^79^, ^80^ This technology superimposes 2D patterns to form a 3D structure. 3D printing procedures typically involve modeling, slicing, printing, and post-processing (Figure 5A).^81^ It has been reported in recent years that complex 3D architectures can be generated from a variety of materials, such as polymers and NPs. Among the materials that can be used for 3D printing, NPs (for example, nanowires, graphene, and QDs) are the most suitable for further research because the assembly arrangement of NPs has great potential to improve structural properties. The electric, light, and magnetic fields are the main driving fields for adjusting the state of the NPs. Additionally, NPs with different morphologies have been designed to improve the 3D printing resolution and manufacturing speed. For example, zero-dimensional (0D) QDs in printed structures affect the visibility or color of the pattern. 1D nanowires in a specific printing direction can improve the electrical conductivity. 2D nanosheets can transform the surface tension of topological structures, thus changing the wettability.^11^ This section analyzes the principles and development prospects of 3D printing assembly technology, and further describes how to drive the alignment of NPs in 3D printing assembly by means of light, electric, mechanical, and magnetic fields. The 3D printing assembly methods for the arrangement of NPs are mainly divided into three categories: stereolithography (SLA) based on reduction polymerization, deposition molding based on extrusion printing, and powder bed fusion (PBF).

#### SLA-based 3D printing assembly

SLA is a 3D printing method based on reductive polymerization using liquid photosensitive polymers, such as photosensitive resins, as consumables. The photocured resin is photopolymerized layer by layer using photo stimulation (Figure 5B).^82^^,^^83^ Optical stimulation is mainly achieved by the interaction of visible light, ultraviolet (UV) light, and laser. SLA technology has the advantages of high material utilization and high accuracy. However, the preparation process is time consuming and complicated and is mainly applied in medical devices, ceramics, and molds.

#### Extrusion-based 3D printing assembly

There are several extrusion-based 3D printing assembly technologies, including direct ink writing (DIW), fused deposition modeling (FDM), direct inkjet printing, electrohydrodynamic printing (EHD), and binder printing. The desired configuration for DIW print assembly technology is obtained by the computer-aided positioning of patterns, pneumatic extrusion of NP inks, and evaporative assembly of NPs under natural conditions (Figure 5C).^84^ DIW technology is suitable for a wide range of ink viscosities (2^6^–10^6^ mPa s), and can print viscoelastic or shear dilution inks with high NP contents. This technology is widely used in the preparation of biological, medical, sensor, and other equipment.^85^

FDM technology produces the target 3D structure by heating and printing thermoplastic filaments (Figure 5D).^83^ Materials that can be printed using FDM technology generally have good fluidity in the molten state, low shrinkage, and rapid cooling molding.

In direct inkjet printing, suspended NP or polymer ink is deposited by electrostatic force and heat, and then solidified by UV light, physical cooling, or chemical reaction. This technology is applicable to a small range of ink viscosities (2–10^2^ mPa s), which makes it difficult to print inks with a high NP content.^86^^,^^87^ DIW, FDM, and direct inkjet printing can be integrated with multiple nozzles to simultaneously print different materials.

EHD is a high-resolution printing method that relies on the voltage between the nozzle and the substrate to spray ink (Figure 5E).^88^ By adjusting the surface tension, electrostatic force, and viscosity of the charged ink droplets on the nozzle, the morphology of the ink can be adjusted to become individual droplets (e-jet printing) or filaments (electrospinning).^16^ Unlike DIW or EHD printing, binder printing coats small molecules on the NP surface to bind them together.^89^

#### PBF-based 3D printing assembly

PBF printing mainly includes selective laser sintering (SLS), selective laser melting, and electron beam forming. Metal powders are the main consumable materials in this technology. The 3D structure is constructed by electron beam or laser selective sintering powder, and then layer-by-layer assembly.^90^^,^^91^ PBF technology has the advantages of high precision and good performance, and is used to prepare fine parts. However, this technology also faces some challenges, such as a complex powder manufacturing process, high requirements for particle size (generally less than 20 μm), low processing efficiency, high cost, and small printing size.^92^

#### Driving force for 3D printing assembly

NPs assembled by 3D printing technology have unique properties; that is, the assembly process is adjustable and points are formed on a surface followed by layer-by-layer printing, functionally enhancing or adjusting 3D devices from the micro- to macroscale. The use of photoelectric, mechanical, and magnetic forces to change the arrangement and performance of NPs during the 3D printing assembly is discussed.

**Figure 6:**
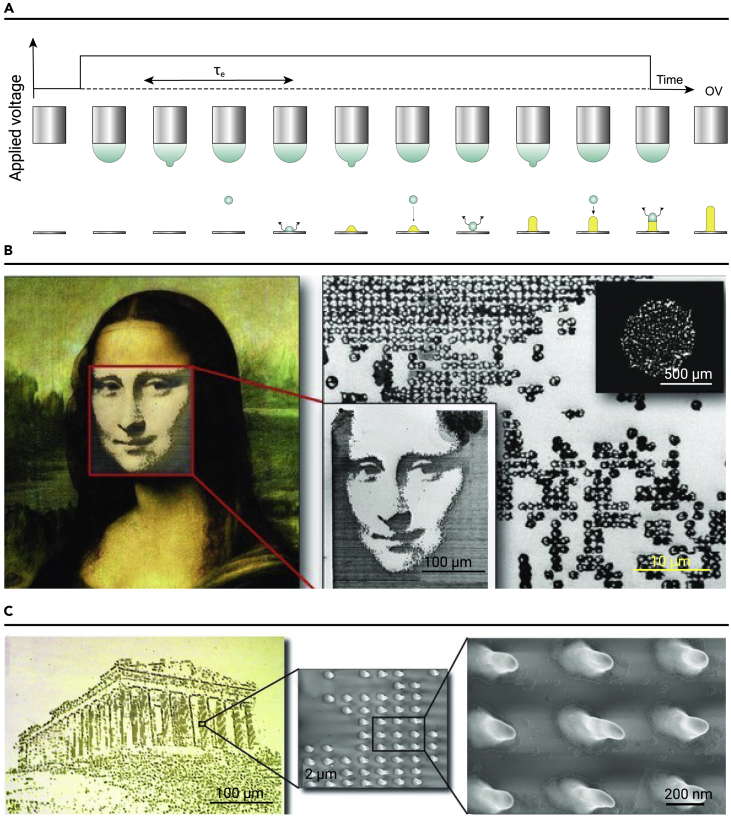
NP patterning based on 3D printing (A) Schematic diagram of the growth process of single nanorods using modified EHD printing. (B and C) Optical photographs and partially magnified SEM images of the face of (B) Mona Lisa composed of AuNPs, and (C) the Parthenon temple composed of ZnO NPs, respectively. Reprinted with permission from Schirme et al.^94^ (copyright 2010, John Wiley & Sons) (B and C).

Electrical stimulation can affect the assembly and arrangement of charged NPs by changing the charged state of the electrically active NPs, inducing electrostatic interactions or producing thermal effects. These interactions change the assembly structure and improve the device performance. Poulikakos et al. modified EHD printing by combining electrohydrodynamic injection with electrostatic nanodroplet autofocusing effects (Figure 6A).^93^ Ink droplets periodically spray individual, micrometer-sized spherical droplets from the nozzle. The tips of the AuNPs formed by the first few droplets act as sharp electrodes, generating a strong electric field gradient and focusing the subsequent assembly of the AuNPs. The diameter of the scaffold was equal to that of a single ink droplet. The structure was influenced by the soft landing hydrodynamics, solvent evaporation rate, and self-assembly of the colloidal building blocks. However, the increase in height along the z axis was caused by intensification of the local electrostatic field. The structure could be grown by additive deposition to form a 3D structure with a large aspect ratio of 50 nm in diameter and up to 850 nm in height.

Optical NPs (such as gold and silver) can enhance the intensity of the localized electromagnetic (EM) field by SPR. Various high-performance optical devices have been fabricated using 3D printing inks (resins and polymers) containing optical NPs. For example, the protein-coupled AuNPs were encapsulated in hydrogel fibers, which significantly enhanced the optical transport through the SPR effect, and greatly improved the sensitivity for the targeted detection of severe acute respiratory syndrome coronavirus-2 (SARS-CoV-2) RNA using resonance shift.^95^ Similarly, light stimulation can induce the assembly and growth of NPs. Poulikakos et al. adopted e-jet technology to print the face of the Mona Lisa on a substrate using colloidal AuNP suspensions with a diameter distribution of 3–7 nm^94^ Figure 6B presents optical photos and a partially magnified scanning electron microscopy (SEM) image of the pattern. This technique can be applied to other NPs, and zinc oxide (ZnO) NPs can be deposited on silicon substrates. A long pulse intensity promoted the growth of nanopillars. Figure 6C shows optical photographs and SEM images of the Parthenon constructed with ZnO NPs.

The fluid shear force in the fluid motion can alter the interaction between NPs, resulting in anisotropic NPs that adjust the assembly process and rearrange along the printing principal axis. By adjusting the force conditions of thermodynamic NPs during printing, 3D-printed devices display unique shape and optical, thermal, and electrical properties. For example, Yang et al. manipulated the shear rate to align and assemble graphene nanosheets during the 3D printing process, which enhanced the mechanical and electrical conductivity of the assembled structures.^96^

Under a magnetic field, magnetic NPs not only can affect the assembly process during 3D printing but can also provide directional transportation and remote control to the assembled devices. Sitti et al. prepared magnetic double-helix cell microtransporters by the 3D printing assembly of polymer inks containing superparamagnetic FeO NPs under uniform magnetic field conditions.^97^ Driven by a rotating magnetic field, transporter-loaded cells were precisely delivered to the target. Chen et al. quickly and efficiently produced a bionic microneedle array by using a magnetic field to drive the assembly and arrangement of FeO NPs in polymer inks during 3D printing. The microneedle array exhibited excellent stability and is expected to be used for painless drug delivery in clinical treatments.^98^

3D printing as a programmable method can directly print an entire product by regulating the computer program with a short process cycle, considerably lower probability of defective products, low cost, and significantly higher functional integration than traditional manufacturing technology. The advantage of 3D printing is that NPs assemble with nanoscale alignment accuracy, improving printing accuracy and realizing the precise manipulation of nanomaterials.^99^^,^^100^ Although 3D printing assembly has been applied in various fields, there are still some limitations. NP alignment based on 3D printing is limited to the 2D x-y plane. Therefore, the main problem with this technology is the NP configuration management along the z axis in the multi-layer preparation process.^19^^,^^90^ Additionally, improving the efficiency and speed of high-precision 3D printing is a research hotspot.

### NP assembly based on 4D printing

4D printing is a manufacturing technology that allows structures to change their shapes or properties under external excitation. The deformation design of the structure is directly incorporated into the filler. Physical or chemical forces, such as thermal, optical, electrical, mechanical, magnetic, and reactants, are used as driving forces.^16^, ^101^ Typically, smart materials for 4D printing exhibit self-sensing, self-actuating, and self-healing properties. Materials are mainly divided into categories according to the deformation driving force of the structures, namely, heat (for example, shape memory polymers [SMPs]),^102^ light (e.g., photoresponsive polymers),^93^^,^^103^ electric fields (e.g., carbon nanotube),^104^, ^105^, ^106^ magnetic fields (e.g., Fe_3_O_4_ and FeO),^107^^,^^108^ and reactants (e.g., polyacrylic acid, poly(n-isopropylacrylamide), and polyvinyl alcohol).^109^, ^110^, ^111^ Although the technology is in its infancy, 4D printed technology combined with NP assembly can serve as an effective method for constructing stimuli-responsive microstructures for reversible and two-way self-assemblies.^112^ This section briefly discusses the application and development prospects of nanoassembly and 4D printing under different stimuli, such as thermal, magnetic, and reactants.

#### Thermally driven nanoassembly and 4D printing

The glass transition temperature is considered as the critical driving point. Owing to the different phases and stress states of the devices above and below the glass transition temperature, the deformation and assembly processes of the devices depend on temperature. Xie et al. successfully developed a crystalline assembly pattern by printing ink on SMP films using lasers. The crystallinity assembly process was controlled by local temperature, which could be regulated by the photothermal effect of the ink. The patterns were deformed and assembled into pyramids, jellyfish, snails, and other shapes at one step above the glass transition temperature and were restored after heating, realizing pixelated memory deformation assembly.^111^

#### Magnetically driven nanoassembly and 4D printing

Magnetically driven nanostructures are obtained by embedding magnetic NPs (for example, iron) into the filler. The deformation assembly process for a structure containing magnetic NPs is programmed according to the type, intensity, direction, and frequency of the magnetic field. Nuzzo et al. fabricated a soft biological structure using DIW printing and assembled a hydrogel containing FeO NPs. The assembly deformation process was determined by the local magnetic field.^108^

#### Reactant-driven nanoassembly and 4D printing

The pH, charged state, or degree of crosslinking of materials often vary with the degree of reaction of the material with the reactants. Therefore, the 4D printing assembly process is regulated by the reactants. Shu et al. fabricated fully aligned mesomorphic structures using SLA-printed liquid crystal elastomers.^111^ Toluene was used to change the crosslinking degree of the structure and to control the assembly process of the mesomorphic arrangement. The prepared structure exhibited high stability and could switch between temporary and fixed structures. This method shows potential to create large ordered reversible assemblies, which are difficult to achieve using conventional assembly methods.

Devices based on 4D printing assembly, compared with traditional devices, are more intelligent, controllable, and occupy less space. This technology is expected to be widely used in microrobots, aerospace, military color-changing equipment, telemedicine, and other fields. Gu et al. demonstrated the basic principle of controllable self-assembly using 4D-printed miniature butterfly wings, successfully realizing reversible and two-way self-assembly for 4D-printed microstructures, which is difficult to obtain by 3D printing alone.^101^ 4D printing assembly still faces many challenges. For example, there are few materials with stimulating responses, and the prepared devices have low adaptability to extreme environments (such as high and low temperature, and corrosive environments). Additionally, the precision, size, efficiency, dynamic driving mode, reversible deformation degree, and deformation process control of the prepared devices are limited.

### Applications

A variety of NPs ranging from 0D nanospheres, 1D nanowires, and 2D nanosheets have been prepared and used to assemble and construct functional devices. NPs play various roles in these devices, such as optical sensing elements, signal amplifiers, conductive layers or electrodes, and electron or hole transport layers. This section reviews the specific applications of NP patterning based on printing assemblies in nanosensing, energy-storage devices, and photodetectors.

#### Nanosensing

With the development of nanotechnology, nanosensors are being gradually implemented in medical care, military, and environmental monitoring. The two key parameters of nanosensors are high throughput and ultrahigh sensitivity.^112^ High-throughput sensors usually depend on multi-channel constructions to achieve multiple responses.^113^ Functional NP assemblies for sensing applications have been demonstrated to have ultrahigh sensitivity to environmental responses.^114^, ^115^, ^116^, ^117^, ^118^ Recent advances in functional devices in conjunction with printing technology for physical, chemical, and biological stimulation that are expected to be ideal candidates for next-generation sensors are highlighted below.

Metal nanomaterials with unique optical, magnetic, and electrical properties have gradually become an irreplaceable part of advanced devices.^117^^,^^118^ Gold and silver NPs are the most widely used NPs because of the SPR effect, which can generate a strong localized electric field, promoting the interaction between NPs and analytes.^117^^,^^118^ Additionally, the SPR effect can induce fluorescence, infrared, and Raman enhancement spectra.^119^, ^120^, ^121^, ^122^ Mazali et al. constructed a paper-based SERS platform using NP ink printing, and a very low analyte concentration of 2 μL could be detected.^123^ There was a good linear relationship between the SERS intensity and analyte concentration. Additionally, the sensor has good repeatability and high stability.

**Figure 7:**
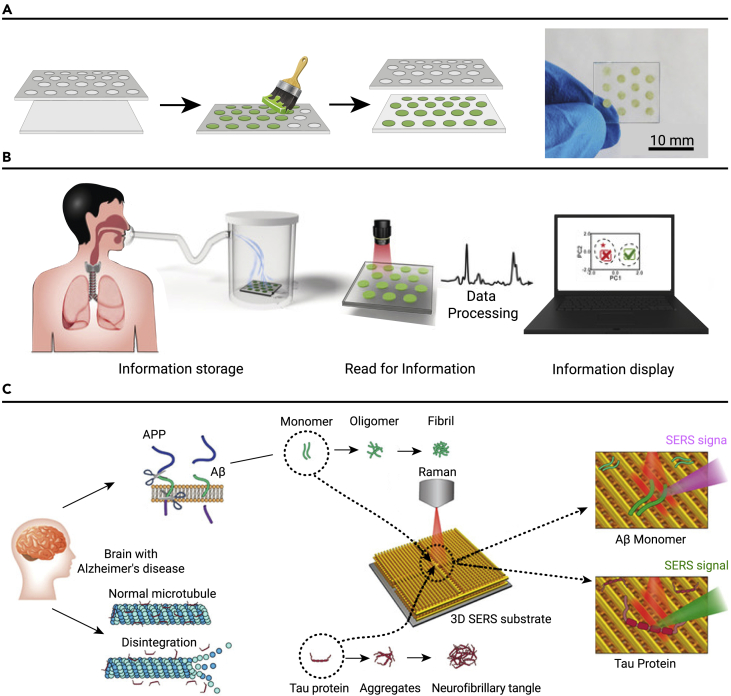
NP patterning based on printing assembly in nanosensing (A and B) (A) Fabrication process of a SERS microarray sensor, and (B) its application in VOC detection. (C) SERS spectra with various concentrations of tau protein and Aβ protein measured by carboxylic acid-functionalized and graphitic nanolayer-coated 3D SERS substrate. Reprinted with permission from Qiao et al.^124^ (copyright 2019, John Wiley & Sons) (A and B), and Park et al.^125^ (copyright 2020, American Chemical Society) (C).

Screen-printing assembly, compared with inkjet printing assembly, is more compatible with the physicochemical properties of inks and the sizes and types of NPs in them. MOFs have the ability to capture guest molecules because of their porosity, and are commonly used for volatile organic compound (VOC) sensing. Our group reported the incorporation of hollow cobalt-nickel layered MOF nanocages on silver nanowires into an array by screen-printing technology.^124^ This array exhibited an excellent gas absorption performance and could detect human VOCs, as shown in Figure 7A. The team used Raman B, G, and R values to produce a unique barcode that could detect aldehyde concentrations. Smart devices (such as smartphones, smartwatches, and scanners) can quickly read information in these barcodes, enabling visual sensing for early disease diagnosis (Figure 7B).

Polymer NPs have been widely used as building blocks for the assembly and formation of ordered periodic structures, which are called photonic crystals (PCs), with photonic band gaps (PBGs).^126^^,^^127^ If the PBG falls within the visible light range, the corresponding structural color is displayed on the surface.^128^ The PC structural color can be easily altered by adjusting the material or diameter of the NPs and the incident angle of light.^129^ Inkjet technology facilitates the alteration of the designed image. Keller et al. printed polystyrene NPs on silicon, glass, and paper substrates.^130^ The three-color palette for surface printing exhibited angular dependence and high visibility, making it legible even in daylight. Using an elaborate design, a simple pattern with red flowers and green leaves was constructed using two NPs with different particle sizes. Patterns responsive to environmental changes were constructed using stimulation-responsive polymer NPs and showed different colors in different gas concentrations.^131^ This gas sensor responded up to 100 times faster than conventional PC sensors owing to the small volume of the printed ink droplets. However, due to the problems of substrate infiltration and ink properties, it is difficult to prepare large-area NP sensors by inkjet printing.

Upconversion nanophosphors (UCNPs), composed of host materials and luminescent centers, have attracted considerable attention owing to their anti-Stokes luminescence properties. Researchers are currently extending the application of UCNPs from the traditional fluorescence field to a new photoelectrochemical field. Chen et al. developed a NIR photochemical sensor based on UCNPs and flower-like WO_3_-modified screen-printed electrodes for the detection of okadaic acid (OA).^132^ By matching the absorption of WO_3_ with the emission of UCNPs, an electron-hole photocurrent is generated by the *in situ* excitation of WO_3_. Under optimal conditions, the 50% inhibitory concentration of the immunosensor reached 0.09 ng mL^−1^. There was a linear relationship between the OA concentration and the antibody binding rate in the range of 0.01–60 ng mL^−1^, and the detection limit was 0.007 ng mL^−1^. The performance is much better than that of traditional kits.

2D nanosheet materials (such as graphene and MXene) have excellent electrical conductivity and large specific surface area, so they are widely employed in electrochemical sensing. Claussen et al. reported high-resolution patterned graphene films (linewidth as low as 20 μm) prepared with inkjet technology.^133^ This film was laser annealed and deposited by platinum NPs, and achieved the electrochemical sensing of hydrogen peroxide with a response time as low as 5 s, a wide linear sensing range of 0.1–550 μm, a sensitivity of 0.21 μM/μA, and a low detection limit of 0.21 μM. This proves that the patterning technique can be combined with electrochemistry to produce various excellent electrochemical devices.

Compared with inkjet printing assembly, required 3D structures can be obtained by a multi-step μCP assembly. Jung et al. constructed a 3D carboxylic acid-functionalized SERS substrate through a multi-step μCP assembly.^125^ The structure was composed of gold nanowire arrays in a crosswise arrangement, which generated a reproducible and strong local EM field. The substrate could measure conformational changes and determine protein concentration by Raman spectroscopy (enhancement factor [EF] = 5.5 × 10^5^). Using the principle, the SERS spectra of the Alzheimer’s disease biomarkers, tau protein and amyloid β protein, were successfully measured and the corresponding secondary structure changes were quantitatively analyzed (Figure 7C). This study proved that SERS had the ability of quantitative analysis of structural changes and early diagnosis of diseases. However, the process is complicated and tedious.

In addition to 2D printing, nanosensors assembled by 3D printing also have great advantages. Adelung et al. developed an acetone sensor based on a mixture of semiconductor metal oxides by DIW assembly.^134^ The device was manufactured by directly writing metallic NPs (iron and copper NPs) onto the target substrate, followed by thermal annealing to form a bridged polyphase semiconductor oxide network. The gas sensor was selective to acetone vapor with a high gas response of approximately 50%, and the lowest operating power was approximately 0.26 μW to 100 ppm.

#### Energy-storage devices

**Figure 8:**
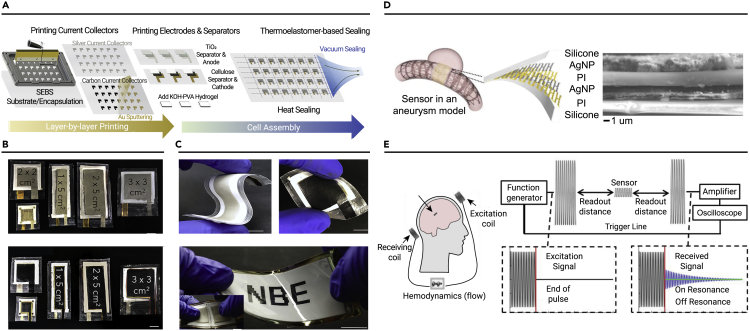
NP patterning based on printing assembly in energy-storage devices (A) Schematic diagram of the screen-printing assembly printing and vacuum-sealing process for AgO-Zn battery cell. (B and C) (B) Photographs of the assembled batteries with different custom sizes, and (C) photographs of the flexible AgO-Zn battery used to drive flexible E-ink display systems. (D) Schematic diagram and cross-sectional SEM image of an implantable flow sensor in an aneurysm model manufactured by inkjet-based 3D printing. (E) Operational schematic diagram and example of received signal for a battery-free wireless implantable flow sensor. Reprinted with permission from Yin et al.^136^ (copyright 2021, Elsevier Inc.) (A–C), and Herbert et al.^138^ (copyright 2019, John Wiley & Sons) (D and E).

Given the popularity of mobile electronic devices, the demand for efficient and low-cost energy-storage devices will continue to increase. Therefore, NP assembly printing technology with simple preparation, high integration, and excellent electrochemical performance is undoubtedly one of the best choices. By changing the properties of inks and substrates with solvent, stabilizer, and NP content, controllable size and shape of NP electrochemical patterns can be obtained on different substrates.^135^ Wang et al. developed a printable polymer AgO-Zn battery using screen-printing technology that exhibited desirable properties such as low impedance, high capacity, good flexibility, and charge-discharge capability.^136^ The deformable, high-throughput, layered screen-printing technology can be used to manufacture elastomer composites, current collectors, electrodes, and separators, as shown in Figure 8A. The size and capacity of the battery were customized with a maximum area capacity of 54 mAh/cm^2^ (Figure 8B). The battery could be used in flexible E-ink display systems and exhibited a better performance than commercial lithium batteries under the same pulse-discharge conditions (Figure 8C). This strategy can provide power for diverse electronic products and will benefit the preparation and application of high-performance retractable batteries. Xue et al. loaded AuNPs onto a 3D conductive skeleton using inkjet printing. Silver NPs, as heterometal seeds, formed zincophilic alloys with Zn, which not only improved the thermal conductivity of the carbon matrix but also guided the uniform nucleation of Zn and avoided dendrite growth, thus obtaining a high-temperature-resistant and foldable Zn battery.^137^

A high degree of integration is essential for portable wearable smart products. Yeo et al. fabricated stretchable wireless electronics using inkjet-based 3D printing (Figure 8D).^138^ The printing process involved the direct microstructural patterning of silver NPs and polyimides. The sensor can be deployed by a catheter and inserted into a blood vessel with an extremely low profile. Wireless inductive coupling enables the wireless analysis of the hemodynamics of bionic brain aneurysms with a maximum reading distance of 6 cm across the flesh (Figure 8E). This study demonstrated the potential of printed biological systems for the battery-free, real-time wireless monitoring of cerebral aneurysm blood flow. Compared with traditional processes, assembly technology is fast and scalable. However, the life span and biocompatibility need to be further improved. Fan et al. first constructed a wearable self-powered sensor on a flexible plastic substrate for ethanol/acetone detection.^139^ The wearable wristband consisted of printed silver interconnects, amorphous silicon solar arrays, MnO_2_-based supercapacitors, and SnO_2_ gas sensors with light-emitting diodes (LEDs). A solar cell powered the sensor and drove the LED indicator to alarm. The supercapacitors acted as photovoltaic energy-storage units to provide power to functional devices during intermittent lighting. The supercapacitors printed with mixed ink provided a high surface capacitance of 12.9 mF cm^−2^, a high specific energy density of 4.5 mWh cm^−3^, and a power density of 7.2 W cm^−3^. This study demonstrated the applicability of the printing assembly method for continuous equipment manufacturing and system integration.

Screen-printing assemblies have shown great potential for the construction of wearable intelligent electronic products through the patterning of inorganic materials and polymers. Recently, Wu et al. demonstrated that printable MXene inks can be used to create MXene-based supercapacitors and lithium-ion batteries.^140^ The supercapacitors connected in series provided a record voltage of 60 V. The quasi-solid batteries exhibited a surface energy density of 154 μWh cm^−2^. Additionally, the fully flexible self-powered system was prepared by integrating the solar cell, lithium-ion batteries, and MXene hydrogel pressure sensors. The integrated system responded to body deformation (i.e., bending of the finger and elbow and pressing vertically) in only 35 ms. Zhang et al. prepared a micro-supercapacitor using a monolayer MXene nanosheet through screen printing.^141^ An area capacitance of 158 mF cm^−2^ and an energy density of 1.64 μWh cm^−2^ was achieved, which is significantly better than other MXene-based capacitors.^8^ Wu et al. constructed a flexible supercapacitor by screen printing. Reduced graphene oxide and manganese hexacyanoferrate active materials were used as inks. The device exhibited the characteristics of on-demand design, bending resistance, high energy density, and strong stability, providing a simple, highly efficient, low-cost, and feasible method for the preparation of high-performance wearable flexible electronic equipment.

#### Photodetectors

Photodetectors convert optical signals into electrical signals, usually in the form of current or voltage.^142^^,^^143^ It is important for photodetectors to be sensitive to light intensity and spectral response speed. In addition to changing material properties, constructing micro-nano arrays is also a very effective method to improve light intensity sensitivity. Tawfique et al. demonstrated the use of black phosphorus as an ink to print photodetectors.^37^ The coffee-ring effect was inhibited by inducing a circulating Marangoni flow and maintaining excellent uniformity. Because of the rapid drying of the ink, the oxidation produced by printing was minimal. The printed device could be used for the passive switching of ultrafast lasers, which remained stable under intense irradiation and was also applied to photodetectors with high response to NIR visibility. Wu et al. obtained a stable 1D α-FAPBI_3_ perovskite structure with high crystallinity and ordered crystal orientation using evaporative lithography assembly.^144^ This 1D structure inhibited trap density and high crystallinity. This photodetector showed an average response rate of 5,282 A W^−1^, an average specific detection rate of more than 1.45 × 10^14^ Jones, and a 3-dB bandwidth of 15 kHz. This patterned technique is only applied to a single system, and restricts the fabrication of multi-materials, heterogeneous structures, and integrated devices. Wu et al. developed an effective strategy for guiding the crystallization of PbI_2_ into microplates in capillary bridges using evaporative lithography assembly.^145^ By controlling the position, size, and orientation of PbI_2_ by capillary bridge, complex patterns such as Latin characters and Arabic numerals can be obtained. The patterns also have an excellent photoelectric performance with a responsivity of 625 mA W^−1^. The technology also allowed the integration of PbI_2_ and poly(3-hexylthiophen-2,5-diyl) (P3HT) for organic/inorganic heterojunction arrays to further improve device performance. The construction of the patterned structure not only improved the photoelectric performance but also further controlled the structural crystallinity, preferred orientation, and polycrystalline processes.

Homogeneous QD NPs tend to self-assemble to form superstructures, which greatly expands the diversity of patterned structure fabrication. Bao et al. uniformly printed perovskite films using the inkjet printing method, enabling the large-scale manufacturing of multi-channel detector arrays. The X-ray photodetector could detect very low X-ray dose rates, indicating that perovskite QDs are ideal candidates for X-ray detection.^146^ Sargent et al. developed an efficient and sensitive PbS QD photodetector using the inkjet printing method.^147^ The designed colloidal ink was stable within an ejectable window without affecting surface passivation. Moreover, photodetectors obtained using this strategy exhibited the highest specific detection rates reported to date (above 10^12^ Jones in NIR). Shorubalko et al. constructed an infrared photodetector using the EHD method to print colloidal PbS QDs onto graphene field-effect transistors.^148^ The technology has accurate positioning and high resolution, and is suitable for manufacturing micro-photodetectors. Notably, the responsivity of the photodetector reached at least 10^9^ Jones at 1,200 nm. The responsivity of the device can be increased by thickness up to 130 nm without affecting the noise current. Most inorganic non-metallic printable materials have the advantages of controllable deposition and chemical stability. However, fluidity issues exist, including fluidity, non-clogging, and storable dispersity.

Semiconductor/metal binary systems are beneficial for obtaining an excellent photoelectric response and device performance. However, the charge diffusion and semiconductor channel mismatch lead to poor carrier transmission, hindering the realization of printed optoelectronics. Song et al. fabricated transverse semiconductor/metal heterostructures with size-matched charge diffusion channels through evaporative lithography assembly.^149^ NPs were self-assembled onto semiconductor nanowires with different morphologies to achieve a high-resolution semiconductor/metal heterogeneous interface. The printed photodetector array showed a high photoelectrical performance with a response sensitivity of 3.41 × 10^12^ Jones and a bending responsivity of 12.9 A W^−1^.

## Summary and prospects

This review discusses advances in NP patterning based on a combination of printing techniques and assembly processes. The various approaches have their respective advantages, but none of the techniques are a panacea for the patterned manufacturing challenges. Table 1 summarizes the available NP materials and the advantages and disadvantages of various methods. 2D printing assembly technologies are suitable for fabricating simple flat patterns. 3D printing assembly technology is used to construct complex devices with localized functional differences. 4D printing assembly technology can satisfy the requirements for the construction of drivable devices. High-resolution patterns have been produced using evaporative lithography, DPN, SLS, SLA, and EHD. Inkjet, screen, and DIW printing have the characteristics of low cost and rapid assembly. Inkjet and FDM printing, and μCP assembly, are limited by the ink type used. Additionally, DIW and screen printing can be adapted to a wide range of inks.

**Table 1:** Summary of all printing methods described in this review

Dimensionality	Printing assembly method	Advantages	Disadvantages	Material	Reference
2D	inkjet printing	not use templates, high accuracy and uniformity, good spatial solution, fast and simple	inks are limited by NPs size and rheological properties	AuNPs, silver NPs, PS, PbS, perovskite, graphene, black phosphorus	^38^^,^^123^^,^^130^^,^^133^^,^^146^^,^^147^
	screen printing	low cost and fast speed, strong compatibility with printing environment	low resolution, high roughness, and high ink viscosity	MXene, silver NPs, MOF, UCNPs, ZnO	^39^^,^^47^^,^^124^^,^^132^^,^^136^^,^^140^
	NIL	simple and low cost, transfer to various substrates	need for primitive mother-templates	Zn_0.2_Fe_2.8_O_4_, AuNPs	^52^^,^^54^
	μCP	high resolution, flexible transfer to various substrates	substrates generally require chemical modification	AuNPs	^58^^,^^125^
	DPN	high resolution, wide range of applicable ink	pattern area is small, difficult to conversion between micrometer and nanoscale	CdSe, ZnS	^61^^,^^62^^,^^63^^,^^69^
	evaporative lithography	high resolution, flexible transfer to various substrates	need for primitive mother-templates	AuNPs, PbI_2_	^74^^,^^144^^,^^149^
3D	SLA	high resolution, high material utilization rate	the process is complicated and takes a long time	photosensitive resin, graphene, FeO	94, 96, 97
	DIW	wide range of viscosity, can print a variety of materials at the same time	low resolution	FeO, copper NPs, cellulose nanocrystals, perovskite	^94^^,^^108^^,^^134^^,^^138^
	EHD	low cost, high resolution, Materials are widely applicable	the process is complex and inefficient	ZnO, AuNPs, PbS	^88^^,^^95^^,^^98^^,^^148^
	PBF	high resolution, high performance	complex preparation, high cost, low efficiency, high particle size requirements	carbon NPs	^90^^,^^91^
4D	thermally driven	simple operating conditions, wide range of materials, high sensitivity	poor adaptability to extreme environments (high temperature, low temperature)	SMP	^101^
	magnetic drive	remote control, good biocompatibility, fast response	there are many influencing factors and the process is complicated	Fe_3_O_4_, FeO	^107^^,^^108^
	the reactants	fast response, wide range of materials	selectivity and accuracy need to be further improved	nanofibers, liquid crystalline	^111^

The variety of functional NPs has become increasingly diverse, resulting in NP-based patterned arrays with more multifunctional properties. Applications of nanopatterned structures are still in the exploratory stage. These fields often require powerful methods and theories to construct high-performance structures. For example, there is no accepted explanation for how scaffold porosity and geometry influence cell adhesion, migration, growth, and differentiation, and how the properties of scaffold structures influence tissue growth.

NP patterning tends to favor applications with complex chemical and topological properties. For example, the PBG of topological PCs is always in the terahertz and gigahertz bands because its structural period is significantly large.^150^^,^^151^ Topological nanostructures are expected to be constructed in the visible and NIR wavelengths using the printing assembly method.

The difficulty of printing assemblies lies in balancing the low cost, high resolution, and high speed, which are simultaneously indispensable but difficult to achieve. For example, inkjet printing can quickly and cheaply produce patterns; however, the patterning resolution is compromised. Therefore, an in-depth understanding of the mechanism of the interaction between ink droplets and substrates with different properties will be beneficial to precisely control the ink printing process. Furthermore, a hybrid strategy using different patterning techniques is necessary to achieve cost-effective patterning.

The main application for printing assembly NP arrays is currently restricted to the laboratory environment owing to the complexity of these methods. However, with the development of technology, low-cost, high-precision, high-performance NP assembly technology will eventually be applied to industrial production. Additionally, the aggregation of NPs has certain negative effects. For example, high ionic strength often reduces the stability of samples. Under ligand exchange, NP aggregation leads to performance degradation.^152^ Meanwhile, when NPs aggregate, the small specific surface area leads to fewer catalytic sites, thus reducing the catalytic performance.

The further development of new functional nanoinks is also a major problem facing 3D printing technology. 3D printing assembly requires precise control of vertical growth through ink drops and pre-printing layers, but the process is difficult to control.

In addition to currently reported methods, there are many other creative alternatives. It is possible to form large areas of more complex patterns, which are stable and compatible with the substrates, on demand. Devices with complex structures and diverse functions can be obtained using clever design methods and modes. Therefore, interdisciplinary research will be conducive to the large-scale assembly of on-demand patterns.

## Lead contact website

https://life.tjut.edu.cn/info/1099/1092.htm.
